# Developmental and Environmental Regulation of Cuticular Wax Biosynthesis in Fleshy Fruits

**DOI:** 10.3389/fpls.2019.00431

**Published:** 2019-04-11

**Authors:** Priyanka Trivedi, Nga Nguyen, Anne Linn Hykkerud, Hely Häggman, Inger Martinussen, Laura Jaakola, Katja Karppinen

**Affiliations:** ^1^Department of Ecology and Genetics, University of Oulu, Oulu, Finland; ^2^Norwegian Institute of Bioeconomy Research, Ås, Norway; ^3^Climate Laboratory Holt, Department of Arctic and Marine Biology, UiT the Arctic University of Norway, Tromsø, Norway

**Keywords:** fruit, cuticle, cuticular wax, biosynthesis, regulation, temperature, light, bioactivity

## Abstract

The aerial parts of land plants are covered by a hydrophobic layer called cuticle that limits non-stomatal water loss and provides protection against external biotic and abiotic stresses. The cuticle is composed of polymer cutin and wax comprising a mixture of very-long-chain fatty acids and their derivatives, while also bioactive secondary metabolites such as triterpenoids are present. Fleshy fruits are also covered by the cuticle, which has an important protective role during the fruit development and ripening. Research related to the biosynthesis and composition of cuticles on vegetative plant parts has largely promoted the research on cuticular waxes in fruits. The chemical composition of the cuticular wax varies greatly between fruit species and is modified by developmental and environmental cues affecting the protective properties of the wax. This review focuses on the current knowledge of the cuticular wax biosynthesis during fleshy fruits development, and on the effect of environmental factors in regulation of the biosynthesis. Bioactive properties of fruit cuticular waxes are also briefly discussed, as well as the potential for recycling of industrial fruit residues as a valuable raw material for natural wax to be used in food, cosmetics and medicine.

## Introduction

**FIGURE 1:**
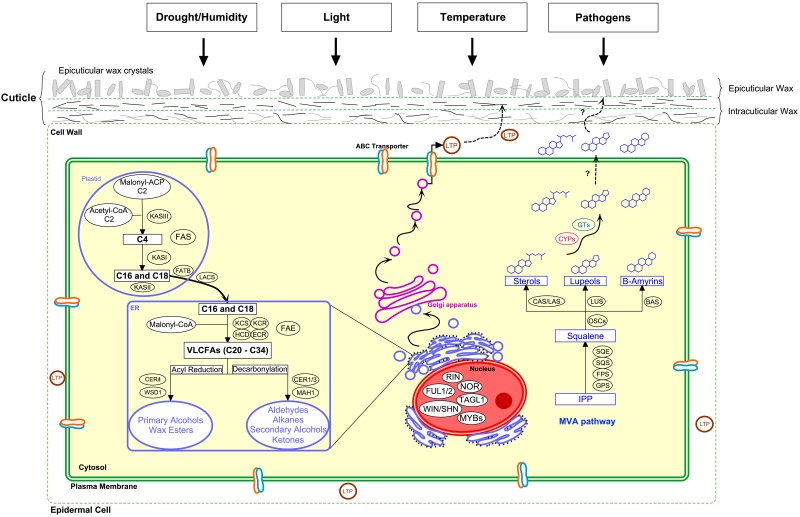
Cuticular wax biosynthesis and interacting environmental factors. Cuticle has an important role as water barrier and in environmental interactions. Biosynthesis of aliphatic wax compounds starts with the generation of fatty acids in plastid by fatty acid synthase complex (FAS). The C_16_ and C_18_ precursors are hydrolyzed by acyl-ACP thioesterase (FATB) and converted to CoA thioesters by long chain acyl-CoA synthase (LACS) before transferred to endoplasmic reticulum (ER). In the ER, fatty acids are extended to very-long-chain fatty acids (VLCFAs) by fatty acid elongase (FAE) complex enzymes β-ketoacyl-CoA synthase (KCS), β-ketoacyl-CoA reductase (KCR), β-hydroxyacyl-CoA dehydratase (HCD), and enoyl-CoA reductase (ECR). VLCFAs are modified to primary alcohols by fatty acyl-CoA reductase (CER4) and further to wax esters by wax synthase (WSD1) through acyl reduction pathway. Decarbonylation pathway produces aldehydes, alkanes, secondary alcohols and ketones by enzymes including fatty acyl-CoA reductases (CER1/3) and a midchain alkane hydroxylase (MAH1). The compounds are transported to the plant surface through Golgi network and ABC transporters and by lipid transfer proteins (LTPs). Wax triterpenoids and sterols are derived from squalene that is produced from isopentenyl diphosphate (IPP) through mevalonic acid (MVA) pathway by geranyl pyrophosphate synthase (GPS), farnesyl pyrophosphate synthase (FPS), squalene synthase (SQS), and squalene epoxidase (SQE). Squalene is cyclized by oxidosqualene cyclases (OSCs) including cycloartenol synthase (CAS), lanosterol synthase (LAS), lupeol synthase (LUS), and β-amyrin synthase (BAS) to produce sterols, lupeols, and amyrins, respectively, which are modified by cytochrome P450 monooxygenases (CYPs) and glycosyltransferases (GTs) before transported to plant surface. TFs important for cuticle development are shown in the nucleus. Modified according to Kunst and Samuels (2009); Sawai and Saito (2011); Lee and Suh (2013); Yeats and Rose (2013); and Thimmappa et al. (2014).

The primary surfaces of aerial parts of land plants are covered by a hydrophobic layer called cuticle. The cuticle is composed of polyester cutin and a mixture of lipidic compounds collectively called wax. The chemical composition of cuticular wax varies between species and organs but is also dependent on the developmental stage and environmental conditions (Yeats and Rose, 2013). Cuticular wax appears as amorphous “intracuticular wax” embedded in cutin matrix, that is connected to the polysaccharides on the underlying epidermal cell walls, and as “epicuticular wax” that may exist as crystallized to various micro-morphologies (Koch and Ensikat, 2008; Fernández et al., 2016; Barthlott et al., 2017; Figure 1). Cuticle not only provides protection against desiccation but also has a role in plant development and environmental interactions (Yeats and Rose, 2013). In fleshy fruits, cuticular waxes have a crucial role in minimizing water loss/uptake through an often astomatous surface, providing mechanical support, preventing fruit softening, and in resistance to pathogens (Saladié et al., 2007; Martin and Rose, 2014; Wang J. et al., 2014). The cuticle in fruits is usually thicker than in leaves and the epicuticular wax is often visible to the naked eye as a white, dull, or glossy coating. Alterations in cuticular wax biosynthesis, load and composition take place during the fruit development to keep it continuous and adjusted to its tasks. From a human perspective, fleshy fruits are an indispensable part of a healthy diet and cuticular wax affects important quality traits for consumers, such as fruit color, texture, shelf-life, sensory and nutritional quality, and preventing fruit cracking (Lara et al., 2014; Petit et al., 2017; Chu et al., 2018a; Tafolla-Arellano et al., 2018).

Recent reviews exist concerning cuticular wax biosynthesis in vegetative organs of plants (e.g., Lee and Suh, 2013; Yeats and Rose, 2013; Borisjuk et al., 2014) but also in fruits mainly focusing on cuticle composition (Lara et al., 2015), genetic regulation of cuticle assembly (Hen-Avivi et al., 2014) and role of cuticle in postharvest quality (Lara et al., 2014). The present review compiles the current knowledge on the developmental and environmental regulation of biosynthesis and composition of cuticular waxes in fleshy fruits.

## Cuticular Wax Composition and Biosynthesis in Fruits

The major components of plant cuticular waxes are very-long-chain fatty acids (VLCFAs, typically C_20_–C_34_) and their derivatives including alkanes, aldehydes, primary and secondary alcohols, ketones, and esters along with secondary metabolites, such as triterpenoids, sterols, tocopherols, and phenolic compounds (Kunst and Samuels, 2009; Yeats and Rose, 2013). The composition of cuticular wax varies widely among fruit species and cultivars (Table 1). While alkanes are common wax components in cuticles of different plant organs, triterpenoids are present especially in fruits (Szakiel et al., 2012). Triterpenoids and *n*-alkanes are the major compounds of cuticular wax in tomato (*Solanum lycopersicum*), apple (*Malus* ×*domestica*), Asian pear (*Pyrus* spp.), sweet cherry (*Prunus avium*), peach (*Prunus persica*), and pepper (*Capsicum annuum*) fruits. Also, among wild tomatoes, alkanes are the dominant compounds but the content of triterpenoids varies between tomato species (Yeats et al., 2012). Instead, the cuticular wax in grape (*Vitis vinifera*), olive (*Olea europaea*), persimmon (*Diospyros kaki*), and blueberries (*Vaccinium* spp.) contain high amounts of triterpenoids but only traces of alkanes (Table 1).

**Table 1:** The main cuticular wax compound classes in various fleshy fruits at mature stage and changes during fruit development.

Species	Main compound classes^∗^	References
Tomato (*Solanum lycopersicum*)	Alkanes (*n*-hentriacontane, *n*-nonacosane) ∼, triterpenoids (amyrins) ∼	Bauer et al., 2004; Leide et al., 2007, 2011; Saladié et al., 2007; Mintz-Oron et al., 2008; Kosma et al., 2010; Petit et al., 2014
Wild tomato (*Solanum* spp.)	Alkanes (*n*-hentriacontane, *n*-nonacosane), triterpenoids (amyrins), esters	Yeats et al., 2012
Eggplant (*Solanum melongena*)	Alkanes (*n*-hentriacontane), alkanoic acids	Bauer et al., 2005
Apple (*Malus* ×*domestica*)	Triterpenoids (ursolic acid) ↓, alkanes (*n*-nonacosane) ↓, primary and secondary alcohols ↑	Belding et al., 1998, 2000; Ju and Bramlage, 2001; Verardo et al., 2003; Legay et al., 2017; Yang et al., 2017; Leide et al., 2018
Asian pear (*Pyrus* spp.)	Alkanes (*n*-hentriacontane, *n*-nonacosane) ↓, triterpenoids (*α*-amyrin) ↑, primary alcohols (triacontanol, triacontane-1,30-diol) ↑, fatty acids ↑	Yin et al., 2011; Li et al., 2014; Heng et al., 2017; Wu et al., 2017, 2018
European pear (*Pyrus communis*)	Alkanes (*n*-hentriacontane), primary alcohols (triacontanol, triacontane-1,30-diol)	Wu et al., 2018
Sweet cherry (*Prunus avium*)	Triterpenoids (ursolic acid) ↓, alkanes (*n*-nonacosane) ↑, fatty acids	Peschel et al., 2007; Belge et al., 2014a; Rios et al., 2015
Peach (*Prunus persica*)	Triterpenoids (ursolic acid, oleanolic acid), alkanes (*n*-tricosane, *n*-pentacosane)	Belge et al., 2014b
Plum (*Prunus domestica*)	Secondary alcohols, alkanes (*n*-nonacosane)	Ismail et al., 1977
Grape (*Vitis vinifera*)	Triterpenoids (oleanolic acid) ↓, alcohols ↓	Radler, 1965; Comménil et al., 1997; Casado and Heredia, 1999; Pensec et al., 2014
Orange (*Citrus sinensis*)	Triterpenoids (friedelin, lupeol) ↑, aldehydes ↑, alkanes (*n*-hentriacontane) ∼, fatty acids ↓	Sala et al., 1992; Liu et al., 2012; Wang J. et al., 2014; Wang et al., 2016
Satsuma mandarin (*Citrus unshiu*)	Aldehydes (octacosanal) ↑, triterpenoids (friedelin) ∼, alkanes (*n*-nonacosane) ∼, fatty acids ∼	Sala et al., 1992; Wang J. et al., 2014
Grapefruit (*Citrus paradisi*)	Triterpenoids (friedelin), aldehydes	McDonald et al., 1993; Nordby and McDonald, 1994
Olive (*Olea europaea*)	Triterpenoids (oleanolic acid) ↓, primary alcohols ↑, fatty acid derivatives ∼	Bianchi et al., 1992; Huang et al., 2017
Persimmon (*Diospyros kaki*)	Triterpenoids (ursolic acid, oleanolic acid), alkanes, alcohols	Tsubaki et al., 2013
Pepper (*Capsicum annuum*)	Triterpenoids (amyrins), alkanes (*n*-hentriacontane)	Bauer et al., 2005; Kissinger et al., 2005; Parsons et al., 2012, 2013
Cucumber (*Cucumis sativus*)	Alkanes (*n*-nonacosane), aldehydes, fatty acids	Wang et al., 2015a,b
Blueberry (*Vaccinium corymbosum*)	Triterpenoids (ursolic acid, oleanolic acid) ∼, β-diketones ↓	Chu et al., 2017, 2018b
Blueberry (*Vaccinium ashei*)	Triterpenoids (ursolic acid) ↑, β-diketones ↓	Chu et al., 2017, 2018b
Cranberry (*Vaccinium macrocarpon*)	Triterpenoids (amyrins), aldehydes	Croteau and Fagerson, 1971
Bayberry (*Myrica pensylvanica*)	Glycerolipids (triacylglycerol, diacylglycerol)	Simpson and Ohlrogge, 2016


*^∗^Proportional change in cuticular wax during fruit development is indicated when information available. ↑, increased proportion; ↓, decreased proportion; ∼ no clear trend. The main compound(s) indicated in parentheses when information available.*

Apart from alkanes and triterpenoids, many fruits have high proportions of other components in their cuticles. A recent study indicated high levels of primary alcohols and tocopherols in the cuticular wax of some pear cultivars (Wu et al., 2017, 2018). Plum (*Prunus domestica*) and some apple cultivars show high proportion of secondary alcohols in fruit cuticle, while tomato cuticle contains significant amounts of polyunsaturated constituents, including alken-1-ols and alkenes (Kosma et al., 2010). Aldehydes are abundant only in cuticles of some fruits, such as cucumber (*Cucumis sativus*), cranberry (*Vaccinium macrocarpon*), and *Citrus* fruits. Cuticular wax of bayberry (*Myrica pensylvanica*) uniquely consists of glycerolipids while blueberries contain high levels of β-diketones (Table 1).

Many of the cuticle properties are affected by the composition of wax. For example, wax composition rather than cuticle thickness has been indicated to affect water transpiration rate (Riederer and Schreiber, 2001). The presence of long-chain alkanes and aldehydes has been found to increase water impermeability of fruit cuticles, while triterpenoids and sterols have opposite effects (Vogg et al., 2004; Leide et al., 2007; Parsons et al., 2012; Wang J. et al., 2014; Moggia et al., 2016). Instead, triterpenoids were shown to enhance mechanical strength of persimmon fruit cuticle by functioning as nano-fillers (Tsubaki et al., 2013). Wax composition also affects epicuticular wax micro-morphology (Koch and Ensikat, 2008). Alkanes, aldehydes and alcohols were shown to promote the formation of epicuticular wax crystals in orange (*Citrus sinensis*) and apples (Liu et al., 2012, 2015; Yang et al., 2017).

The knowledge of cuticular wax biosynthesis has mainly been gained from the studies in *Arabidopsis* leaves, but also from tomato fruit owing to its thick, astomatous, easy-to-isolate cuticle and availability of mutants (Bernard and Joubès, 2013; Lee and Suh, 2013; Hen-Avivi et al., 2014). During recent years high-throughput sequencing has facilitated the identification of candidate genes involved in the fruit cuticle formation and wax biosynthesis in addition to tomato (Mintz-Oron et al., 2008; Matas et al., 2011) in apple (Albert et al., 2013; Legay et al., 2015), mango (*Mangifera indica*, Tafolla-Arellano et al., 2017), sweet cherry (Alkio et al., 2012, 2014), orange (Wang et al., 2016), pear (*Pyrus pyrifolia*, Wang Y. et al., 2014), and bayberry (Simpson and Ohlrogge, 2016).

The cuticular wax components are biosynthesized in the epidermal cells of fruit peel. The biosynthesis of aliphatic wax constituents utilizes C_16_ and C_18_ fatty acids produced by *de novo* synthesis in plastids (Figure 1). These precursors are elongated to C_20_–C_34_ VLCFAs in endoplasmic reticulum (ER) by the fatty acid elongase (FAE) complex with β-ketoacyl-CoA synthase (KCS) as the rate-limiting enzyme of the complex (Kunst and Samuels, 2009; Yeats and Rose, 2013). Tomato *lecer6* mutant has shown that *KCS* plays a key role in wax aliphatic compound biosynthesis and determines the chain-length of VLCFAs in tomato fruit (Leide et al., 2007). The resulting VLCFAs can be converted into primary alcohols and esters by acyl reduction pathway or aldehydes, alkanes, secondary alcohols and ketones by decarbonylation pathway (Kunst and Samuels, 2009). In decarbonylation pathway, *CsCER1* and *CsWAX2* (*CER3*) of cucumber and *PaCER1* of sweet cherry was recently shown to play important roles in alkane biosynthesis (Alkio et al., 2012; Wang et al., 2015a,b), while *CsCER3* was linked to aldehyde biosynthesis in orange fruit (Wang et al., 2016). Also *CsCER4* linked to wax biosynthesis was recently identified in cucumber (Wang W. et al., 2018). Wax triterpenoids and sterols are biosynthesized from squalene produced from mevalonate (MVA) pathway followed by modifications into various compounds (Sawai and Saito, 2011; Thimmappa et al., 2014; Figure 1).

## Developmental Regulation of Fruit Cuticular Wax Formation

Tomato is a model species for studying regulation of fleshy fruit development and ripening (Karlova et al., 2014). During the last decades, intensive studies in tomato performed in cuticle formation indicate connections in regulatory network between cuticle and fruit development. Transcription factors (TFs) NON-RIPENING (NOR), and RIPENING INHIBITOR (RIN) are important regulators of fruit ripening, but tomato *nor* and *rin* mutants also show altered fruit cuticular wax profile from early stage throughout the fruit development (Kosma et al., 2010). In addition, other ripening regulators, including FRUITFULL (FUL1,2) and TOMATO AGAMOUS-LIKE1 (TAGL1), have been linked to fruit cuticle development (Bemer et al., 2012; Hen-Avivi et al., 2014; Giménez et al., 2015).

In climacteric fruits, including tomato and apple, plant hormone ethylene acts to initiate and co-ordinate ripening processes, while in many non-climacteric fruits abscisic acid (ABA) has been shown as ripening inducer (Cherian et al., 2014; Karppinen et al., 2018). Both ethylene and ABA signaling seems to play important roles in fruit cuticle biosynthesis (Ziv et al., 2018). Studies have indicated that ethylene accelerates cuticular wax accumulation in orange and apple (Ju and Bramlage, 2001; Cajuste et al., 2010; Li et al., 2017). The *Arabidopsis* members of the SHINE (WIN1/SHN1) clade of ethylene responsive factors (ERFs), transducing signal from ethylene, are well-characterized regulators of the cuticular wax biosynthesis (Aharoni et al., 2004; Broun et al., 2004). In tomato, *SlSHINE3* (*SlSHN3*) was shown to regulate fruit cuticle formation and cuticular lipid biosynthesis (Shi et al., 2013). Also the expression of sweet cherry, apple and mango homologs for *WIN1/SHIN1* coincided with fruit cuticle deposition (Alkio et al., 2012; Lashbrooke et al., 2015b; Tafolla-Arellano et al., 2017). Downstream to *SlSHN3*, MYB TF *SlMIXTA* has been shown to regulate fruit cuticle assembly in tomato (Lashbrooke et al., 2015a; Ewas et al., 2016). Recently, a grape berry-specific ERF *VviERF045*, resembling SHINE clade members, and *Malus* AP2/SHEN member *McWRI1* were indicated in regulation of cuticular wax biosynthesis (Leida et al., 2016; Hao et al., 2017). A connection between ABA and cuticular wax biosynthesis was demonstrated in orange fruit (Wang et al., 2016). In cucumber, ABA was shown to induce gene expression involved in cuticle alkane biosynthesis (Wang et al., 2015a,b).

Due to the multiple tasks, maintaining intact cuticle over the fruit development is necessary, but challenging, due to rapid and extensive surface expansion. Cuticular wax deposition starts early in fruit development (Comménil et al., 1997; Casado and Heredia, 2001; Curry, 2005; Domínguez et al., 2008). However, the pattern of wax load varies markedly between species (in contrast to cutin load) and indicates separately regulated wax biosynthesis from cutin biosynthesis (Wang et al., 2016). In many fruits, including apple (Ju and Bramlage, 2001; Lai et al., 2016), orange (Liu et al., 2012; Wang et al., 2016), pear (Li et al., 2014), blueberries (Chu et al., 2018b), bayberry (Simpson and Ohlrogge, 2016), and mango (Tafolla-Arellano et al., 2017), cuticular wax load increases during the fruit development leading to a thick cuticle at maturity. Furthermore, in many fruits, modification of the wax chemical profile and cuticle accumulation, even after harvest has been reported (Ju and Bramlage, 2001; Belge et al., 2014a,b; Tafolla-Arellano et al., 2017; Yang et al., 2017). Tomatoes also have a thick cuticle at maturity but there are clear cultivar-specific variations in cuticle development (España et al., 2014). In cherry tomatoes, cuticular wax is deposited early in fruit development (Domínguez et al., 2008), while in medium-sized tomatoes, such as “Micro Tom” and “Ailsa Craig,” the wax amount reaches its maximum level at orange-colored stage (Leide et al., 2007; Mintz-Oron et al., 2008) and in some other cultivars the wax amount increases continuously toward the fruit maturity (Bauer et al., 2004). In tomato, all the wax compound classes, except branched alkanes, accumulate during the cuticular wax load (Leide et al., 2007; Mintz-Oron et al., 2008; Kosma et al., 2010). However, in many cases, the continuous wax load leads to changes in the cuticular wax profile during the fruit development (Table 1). For example, in apple, hydrocarbons and triterpenoids predominate in cuticles of young fruits while fatty acids, alcohols and esters contribute mostly to the wax increase during fruit ripening increasing wax greasiness (Ju and Bramlage, 2001; Yang et al., 2017).

High cuticular wax deposition rate at the early stages of fruit development followed by reduction at later stages has been described for sweet cherry (Peschel et al., 2007; Alkio et al., 2012; Lai et al., 2016) and grape (Comménil et al., 1997; Becker and Knoche, 2012; Pensec et al., 2014). The decrease in sweet cherry wax load toward fruit maturity was mainly attributed to the decrease in triterpenoids (Peschel et al., 2007). Similarly, the total triterpenoids decreased during the development of grape berries (Pensec et al., 2014). The role of cuticle as a mechanical support at fruit ripening is important when degrading cell walls cannot sustain the fruit internal pressure. Thus, the inability of the wax deposition to keep in the pace with surface expansion makes ripening fruits vulnerable for micro- and macro-cracking leading to uncontrolled water movement and fungal infections (Comménil et al., 1997; Børve et al., 2000). Cracking is a serious problem in many fruit species, such as tomato and cherries (Domínguez et al., 2012). Recently, an association between cuticular *n*-nonacosane level and cracking tolerance among sweet cherry varieties was described by Rios et al. (2015). Failure in cuticle deposition associated with micro-cracking can cause formation of russeting, a common disorder in fruits, such as apples and pears (Khanal et al., 2013). Improper cuticular wax deposition was shown to be accompanied by the decreased expression of wax biosynthetic genes and *MdSHN3* TF in russeted apples (Lashbrooke et al., 2015b; Legay et al., 2015, 2017).

## Environmental Regulation of Fruit Wax Biosynthesis and Composition

Being a protective barrier on fruit surface, cuticle has a crucial role in the tolerance to various environmental stresses (Figure 1), including osmotic stress (Shepherd and Griffiths, 2006; Xue et al., 2017). Both drought stress and humidity have been shown to affect cuticle deposition. In general, a decrease in cuticle deposition has been detected in plants under high humidity (Tafolla-Arellano et al., 2018). In tomato fruit, decreased cuticle thickness was detected in high humidity, but had no effect on wax accumulation (Leonardi et al., 1999; Domínguez et al., 2012). Instead, plants adapted to water deficit conditions usually have well-developed cuticles in fruits (Crisosto et al., 1994; Barker and Procopiou, 2000; Xue et al., 2017). Regulation of cuticular wax biosynthesis in response to drought stress has been most intensively studied in *Arabidopsis* but also in tomato and cucumber (Xue et al., 2017). In tomato, overexpression of *SISHN1* TF induced expression of wax biosynthetic genes leading to enhanced cuticular wax deposition and drought-tolerance compared to control plants (Al-Abdallat et al., 2014). In cucumber, the expression of fruit-specific cuticular wax genes *CsCER1* and *CsWAX2* increased under drought and salinity stresses (Wang et al., 2015a,b). Furthermore, transcriptome level studies in drought-sensitive cucumber variety suggested that the decreased expression of cutin, suberin, and wax biosynthetic genes might be responsible for sensitivity to drought (Wang M. et al., 2018).

Both light and temperature can directly change the morphology and properties of fruit epicuticular wax (Schirra et al., 1999; Charles et al., 2008). For example, a post harvest heat treatment at 38°C was shown to affect the structure of the epicuticular wax in apple (Roy et al., 1994). However, temperature changes can also modify the biosynthesis of fruit cuticular waxes. Since wax layer is important in maintaining postharvest quality (Lara et al., 2014; Chu et al., 2018a), most temperature treatments have been performed on postharvest fruits. In *Malus* fruits, low temperature treatment (+4°C) increased the thickness of cuticular wax compared to control fruits and up-regulated the expression of *McWRI1*, *McKCS*, *McLACS*, and *McWAX* leading to the accumulation of alkanes (Hao et al., 2017). Similarly, expression of cucumber fruit-specific *CsCER1* and *CsWAX2* were induced by low temperature (Wang et al., 2015a,b). Changes in fruit cuticular wax content and composition during cold storage have also been reported for blueberries (Chu et al., 2018b), Asian pears (Wu et al., 2017), grapefruit (*Citrus paradisi*, Nordby and McDonald, 1991), and sweet cherries (Belge et al., 2014a).

Cuticle is the first barrier to receive light radiation. The increase in thickness of the cuticular wax layer as a response to higher irradiation has been shown in many plant species (Shepherd and Griffiths, 2006; Tafolla-Arellano et al., 2018). In grape berries, the cuticle amount was reported to be higher in sun-exposed berries compared to berries developed in canopy shade (Rosenquist and Morrison, 1989). Also, the spectral quality of light affects the cuticular wax biosynthesis and several reports show that cuticular wax plays a role in the protection against damaging UV-light. Irradiation with enhanced UV-B or UV-C has been demonstrated to increase total amount of cuticular wax and alter wax composition (Tafolla-Arellano et al., 2018). Monochromatic far-red light was shown to stimulate the cuticular wax biosynthesis increasing hydrophobicity of the wax in both tomato and bell pepper fruits during storage (Cozmuta et al., 2016a,b). In grapefruit and mango, interaction of light and temperature conditions affected fruit cuticle accumulation and cuticular wax composition considering difference between fruits growing in interior or exterior canopy (McDonald et al., 1993; Léchaudel et al., 2013).

## Bioactivity and Commercial Potential of Waxes

Cuticle serves as a primary defense against pathogens and affects susceptibility of fruits to pathogens (Comménil et al., 1997; Saladié et al., 2007; Shi et al., 2013). It was shown in sweet orange and pepper that fruits respond to fungal infections by increasing the cuticle load (Kim et al., 2004; Marques et al., 2012). Agudelo-Romero et al. (2015) reported that grape berries infected with *Botrytis cinerea* accumulated saturated long-chain fatty acids with simultaneous up-regulation of genes related to lipid and wax biosynthesis, including acyl-CoA synthetases (LACSs). A transcriptome analysis of *Colletotrichum gloeosporioides* infected tomato fruits showed activation of genes linked to the formation of cuticular wax VLCFAs (Alkan et al., 2015). Also, a contact of orange fruit with yeast *Kloeckera apiculata* was shown to trigger biosynthesis of cuticular waxes and expression of *CsKCSs* leading to increased wax hydrophobicity and changes in wax morphology (Liu et al., 2014).

In addition to cuticles acting as physical barriers, recent findings suggest that cuticle composition rather than thickness determines fruit susceptibility to pathogens (Reina-Pinto and Yephremov, 2009; Ziv et al., 2018). Fruit cuticular waxes are especially rich sources of triterpenoids, which have clear bioactive properties, such as anticancer, anti-inflammatory, antimicrobial and cardioprotective (Dzubak et al., 2006; Szakiel et al., 2012). He and Liu (2007) isolated triterpenoids from apple peels and reported antiproliferative activity against human cancer cells. The antifungal activity of Asian pear fruit cuticular wax was associated with *n*-alkanes, fatty acids along with triterpenoids (Yin et al., 2011; Chen et al., 2014; Li et al., 2014).

Plant cuticles potentially offer a natural alternative for synthetic waxes. Industrial leftover material in particular, such as peels from juice production, provides raw material for isolating fruit wax compounds. For example, extraction of apple peel pomace using supercritical fluid extraction (SFE) demonstrated the reuse potential of juice industry leftovers as a source for value-added wax (Li et al., 2015). Recently, Tedeschi et al. (2018) demonstrated the utilization of fatty acids from tomato pomace waste for production of packaging films. Thus, fruit cuticular waxes from industrial waste can provide sources for bioactive compounds and biodegradable products for the use in pharmaceuticals, cosmetics, packaging, nanocoatings, and the food industry.

## Author Contributions

All authors (PT, NN, ALH, HH, IM, LJ, and KK) have participated in preparation of the manuscript and have accepted the final version.

## Conflict of Interest Statement

The authors declare that the research was conducted in the absence of any commercial or financial relationships that could be construed as a potential conflict of interest.
